# Biofilm Formation by *Staphylococcus aureus* in the Specific Context of Cystic Fibrosis

**DOI:** 10.3390/ijms24010597

**Published:** 2022-12-29

**Authors:** Vincent Jean-Pierre, Agathe Boudet, Pauline Sorlin, Quentin Menetrey, Raphaël Chiron, Jean-Philippe Lavigne, Hélène Marchandin

**Affiliations:** 1 HSM—HydroSciences Montpellier, Université de Montpellier, CNRS, IRD, Service de Microbiologie et Hygiène Hospitalière, CHU Nîmes, 34093 Montpellier, France; 2 VBIC—Virulence Bactérienne et Infections Chroniques, Université de Montpellier, INSERM U1047, Service de Microbiologie et Hygiène Hospitalière, CHU Nîmes, 30900 Nîmes, France; 3 HSM—HydroSciences Montpellier, Université de Montpellier, CNRS, IRD, 34093 Montpellier, France; 4 INFINITE—Institute for Translational Research in Inflammation, Université de Lille, INSERM U1286, CHU Lille, 59000 Lille, France; 5 HSM—HydroSciences Montpellier, Université de Montpellier, CNRS, IRD, Centre de Ressources et de Compétences de la Mucoviscidose, CHU Montpellier, 34295 Montpellier, France

**Keywords:** *Staphylococcus aureus*, biofilm, cystic fibrosis, multispecies biofilm, anti-biofilm

## Abstract

*Staphylococcus aureus* is a major human pathogen whose characteristics support its success in various clinical settings including Cystic Fibrosis (CF). In CF, *S. aureus* is indeed the most commonly identified opportunistic pathogen in children and the overall population. *S. aureus* colonization/infection, either by methicillin-susceptible or methicillin-resistant strains, will become chronic in about one third of CF patients. The persistence of *S. aureus* in CF patients’ lungs, despite various eradication strategies, is favored by several traits in both host and pathogen. Among the latter, living in biofilm is a highly protective way to survive despite deleterious environmental conditions, and is a common characteristic shared by the main pathogens identified in CF. This is why CF has earned the status of a biofilm-associated disease for several years now. Biofilm formation by *S. aureus*, and the molecular mechanisms governing and regulating it, have been extensively studied but have received less attention in the specific context of CF lungs. Here, we review the current knowledge on *S. aureus* biofilm in this very context, i.e., the importance, study methods, molecular data published on mono- and multi-species biofilm and anti-biofilm strategies. This focus on studies including clinical isolates from CF patients shows that they are still under-represented in the literature compared with studies based on reference strains, and underlines the need for such studies. Indeed, CF clinical strains display specific characteristics that may not be extrapolated from results obtained on laboratory strains.

## 1. *S. aureus*, a Major Pathogen in the Biofilm-Associated Disease, Cystic Fibrosis

*Staphylococcus aureus* is a major human pathogen whose characteristics support its success in diverse clinical settings like bacteremia, skin and soft tissue infections, necrotizing pneumonia, device-associated infections like catheter-related infections and ventilator-associated pneumonia, and Cystic Fibrosis (CF) [1]. Besides a wide panel of secreted virulence factors and its multidrug-resistant capacities, biofilm formation is a significant feature that protects *S. aureus* against host defenses and eradication measures and, consequently, allows it to persist in the host [2,3]. Biofilm is defined as a more or less complex, diverse, three-dimensional structured microbial community embedded in a matrix formed by extracellular polymeric substances (EPS) whose major components are extracellular DNA (eDNA), extracellular polysaccharides and structural proteins. Biofilms were long considered as being attached to a surface. However, more recently, biofilms have also been found unattached to any surface. presenting as three-dimensional aggregates, a form that may be closer to the in vivo situation, particularly in mucosal infections like CF [4,5]. Biofilm formation is a dynamic, coordinated, cyclic process, classically described as involving several stages. It begins with the initial reversible, then irreversible, attachment of planktonic bacteria to a surface, secretion of various matrix-forming EPS and bacterial proliferation with microcolony formation leading to a mature, multilayered biofilm of sessile bacteria. The final stage in the biofilm lifecycle is dispersion, controlled through interbacterial communication such as quorum sensing, during which bacteria detach from the biofilm, return from a sessile to a planktonic stage, and may colonize new sites [1,6]. Biofilm formation is not just a protected way of life: it also represents an additional factor favoring the emergence of resistance towards antimicrobial treatments. Indeed, sessile cells are exposed to sub-inhibitory concentrations of antibiotics due to penetration defects and their close proximity in biofilms facilitated horizontal transfer of resistance-encoding genes. Finally, biofilms also contain a subpopulation of quiescent bacterial cells named persisters that are transiently tolerant to antimicrobial stresses [7]. Living in biofilm thus represents a major advantage for persistence during chronic diseases like CF and this must be considered in the management of biofilm-associated diseases like CF.

Cystic Fibrosis is an inherited disease affecting the CFTR (cystic fibrosis transmembrane conductance regulator) protein, marked by a vicious cycle of infection and inflammation which is highly deleterious for the patient’s lung function. *Pseudomonas aeruginosa* and *S. aureus* are the two major pathogens in CF. Although *P. aeruginosa* is dominant in the adult population, *S. aureus* is the most commonly identified opportunistic pathogen in children and the overall CF population [8] with 60 to 80% of CF patients under 20 years old being colonized according to French and American cystic fibrosis registries [9,10,11]. After initial colonization, a subset of patients, representing 36% of patients according to data from the European Cystic Fibrosis Society patient registry, will become chronically colonized [11]. This bacterial persistence is favored by local impairment in host defenses (for example, altered function of macrophages and antimicrobial peptides) as well as the adaptive faculties of the pathogen to the CF lung, a stressful environment wherein pathogens are subjected to diverse and fluctuant abiotic and biotic selective pressures such as hyperinflammation, oxidative stress, limitations in and competition for nutrients and space, anaerobiosis, increased acidity in airway surface liquid and exposure to antibiotics [12,13,14,15]. Biofilm formation is one of the bacterial adaptive responses to environmental stress and, despite administering suitable antibiotics to CF patients, pathogens may elicit persistent colonization that can be attributable to biofilm formation. Nowadays, CF is considered as a biofilm-associated disease but, due to the unique characteristics of CF airways, the particularities of biofilm formation by the major opportunistic pathogens identified in CF patients warrant further consideration. This will enable us to more precisely decipher the pathogenesis of persistent infections in this particular context, especially as biofilm control is the goal of many antimicrobial strategies [16,17]. However, as far as *S. aureus* is concerned, biofilm formation and the molecular mechanisms governing and regulating it have been extensively studied but have received less attention in the unique context of CF lungs.

## 2. Delineation of Information Gathered from a Review of the Literature and Scope of the Review

**Figure 1:**
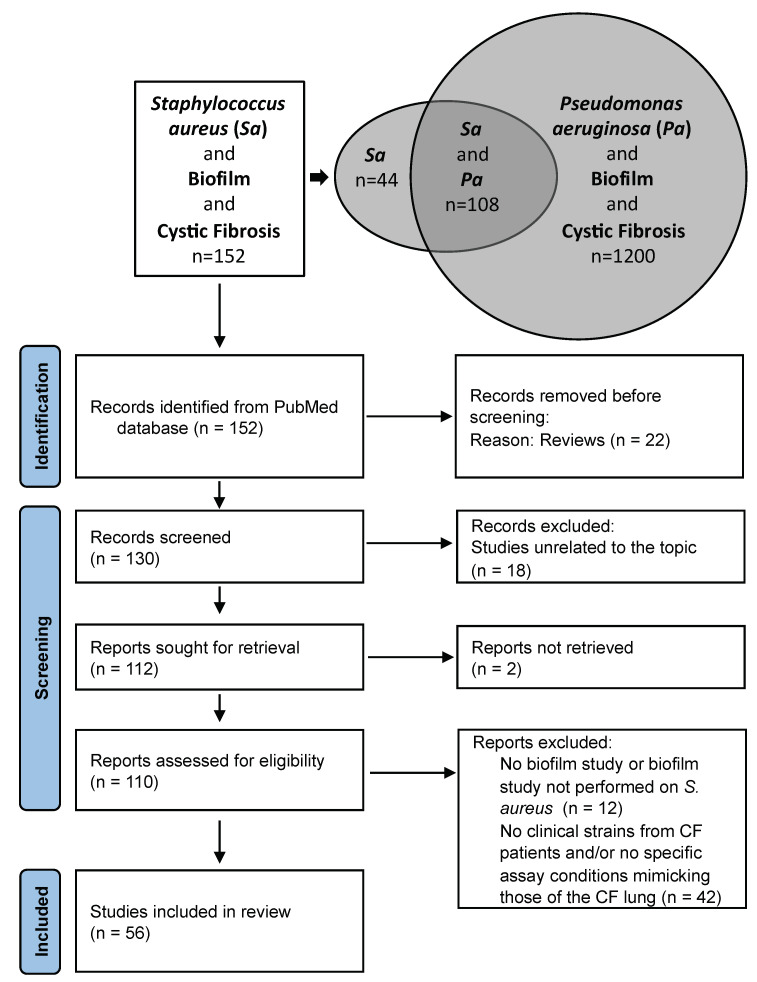
Results of the literature search on *S. aureus* biofilm in the context of Cystic Fibrosis. (PubMed database, search date: 8 October 2022) (**top**) and flow diagram of study selection (**bottom**). *Sa*: *Staphylococcus aureus*; *Pa*: *Pseudomonas aeruginosa*; n: number of publications (not to scale); CF: Cystic Fibrosis.

A literature search using “*Staphylococcus aureus*”, “Biofilm” and “Cystic Fibrosis” in the PubMed database found 152 publications and a similar search for “*Pseudomonas aeruginosa*” found 1200 results and a combined search for “*Staphylococcus aureus*” and “*Pseudomonas aeruginosa*” gave 108 publications (8 October 2022) (Figure 1). This highlighted the fact that studies on *S. aureus* biofilm formation in CF frequently included the two major pathogens, studied either independently or considered through the prism of interactions in dual-species biofilms, a situation closer to the conditions of the CF lung compared to mono-species biofilms. The proportion of patients co-infected by *S. aureus* and *P. aeruginosa* is indeed estimated to be around 30% [18].

The main limitation was identified after reviewing the sections ‘Materials and Methods’ sections of the manuscripts selected, as many articles did not include any clinical strains from CF patients and only studied reference laboratory strains as material to support the observations reported (Figure 1). This was notably observed when reviewing the literature on the consequences of bacterial interactions with *S. aureus* on biofilm formation in CF. Among the *S. aureus* strains most often used in the literature we reviewed were: the community-acquired methicillin-resistant (MRSA) strain USA300 LAC [19,20] and its derivative strain JE2 [18,21] from skin and soft tissue infection; the hospital-acquired MRSA USA100 Tokyo clone [19]; strain 15,981, a strong biofilm-forming strain from an otitis infection [22]; strain ATCC 29,213 (=LMG 10,147) from a wound [23]; and the Newman strain isolated in 1952 from a human infection [24,25]. Some surprising choices of *S. aureus* reference strains were also noted in certain studies. These included strain ATCC 6538, a standard strain for biocide susceptibility testing [26,27] and strain 502A [19], a strain isolated in 1963 from a nurse working in a newborn nursery and further used to colonize infants to prevent colonization by more invasive *S. aureus* strains [28].

The use of these reference strains may not reflect the behavior of clinical CF strains as laboratory maintenance may affect their genomic integrity and alter phenotypic traits over the years [29,30]. Currently there is increasing evidence that clinical and reference strains do not behave identically [31,32,33]. Although studies on reference strains and those including clinical strains provide complementary results, our particular aim was to summarize the knowledge obtained in the specific context of CF. We therefore voluntarily limited the data presented hereafter to those originating from studies on clinical strains isolated from CF patients, with the exception of studies based on reference strains when the latter were studied under specific experimental conditions mimicking those of CF airways (such as growth in a cystic fibrosis mucus-mimicking medium), to more faithfully reflect the in vivo conditions found in CF patients.

## 3. The Challenge of Studying Biofilm Formation in the Context of Cystic Fibrosis

A few of the studies we reviewed reported direct visualization of biofilm by scanning electron microscopy (SEM) and further viability evaluation by confocal laser scanning microscopy (CLSM) combined with Live/Dead stain. These approaches were applied to the toothbrushes of CF children and helped demonstrate that sessile *S. aureus* colonize the toothbrushes of colonized patients and may represent a reservoir for patient reinfection [34]. Interestingly, SEM that allows the study of the spatial structure of biofilm and detects the presence of EPS based on surface scattering and absorption of electrons, showed modifications of the biofilm structuration on toothbrushes of children who had received antibiotic treatment with no more surface bacteria attached to EPS and viable bacteria encased inside thick EPS [34]. Similarly, CLSM that provides three-dimensional images of biofilms showed clear differences in bacterial aggregation, depending on the type of biomaterial commonly used in dentistry [35]. Fluorescence electron in situ hybridization using a specific peptide nucleic acid (PNA-FISH) probe revealed the presence of sessile *S. aureus* on the sinus mucosa of CF patients supporting the fact that both upper and lower airways were the sites of *S. aureus* biofilm formation. In CF patients suffering from chronic rhinosinusitis, the sinuses may thus represent a reservoir for *S. aureus*, predisposing them to recurrent lung infection [36].

However, the majority of currently available studies are based on in vitro approaches to studying *S. aureus* biofilm whereas fewer, more recent, studies used ex vivo lung models. In both conditions, CF mucus-mimicking media, also called artificial sputum medium (ASM) or synthetic CF sputum medium (SCFM), were used in some studies to provide results more relevant to the CF lung than those obtained by using the media normally recommended, i.e., Trypticase Soja (TS) supplemented or not with glucose and NaCl, Brain Heart Infusion (BHI) or Luria Broth (LB) due to rheological properties closer to those of the CF mucus [37,38,39,40,41]. These studies showed that the results depend on the medium in which the biofilm was developed and, broadly speaking, on the overall culture conditions (atmosphere, biofilm substrate, etc.) affecting biofilm growth, metabolic activity and resistance to antimicrobials [37,38]. In particular, Haley et al. using SEM and CLSM to evaluate biovolume, biomass, thickness and roughness of the biofilm showed that these structural parameters varied according to biofilm growing conditions [37]. However, nine artificial mucus medium formulations were successively described that may not provide similar results due to variable composition, either containing mucin, egg emulsion, glucose, or not [37,41]. Similarly, another study used protein-conditioned surfaces, i.e., polystyrene microplates coated with mucin or elastin with the aim to reproduce conditions closer to those found in vivo in CF patients [42]. Finally, one interesting study used experiments based on the timeline of lung colonization, i.e., more generally, a first colonization by *S. aureus* followed by *P. aeruginosa* in CF patients, and demonstrated that introducing the two species according to a timeline, and at various inoculation strengths, affects the formation of biofilm [27].

### 3.1. Biofilms In Vitro Assay Approaches

The ability to form biofilm was evaluated by Bernardy et al. through qualitative phenotypic characterization of exopolysaccharide (EPS) production using Congo red agar plates on a collection of *S. aureus* from CF patients showing strains that did not produce EPS to EPS-overproducing strains, even in the same patient [43]. Besides this screening method, a large panel of methods is available for biofilm growth, characterization and visualization allowing the evaluation of bacterial adhesion, biofilm biomass, viability and/or matrix composition. In addition, the development of fluorescence imaging techniques either after live/dead staining, differential fluorescent staining of species in multispecies biofilms or staining of the biofilm components by specific probes (cells, EPS, proteins, …) allowed high resolution study of the biofilm spacial structure. For a review of all these biofilm study methods and their respective advantages and limitations, see Azeredo et al. [44]. They can globally be classified into static (usually using microplates) or dynamic (under flow or microfluidic) methods. Among them, biofilm biomass quantification based on Crystal Violet (CV) staining in microtiter plates was the most commonly used technique for studying *S. aureus* strains from CF patients (Supplementary Table S1). However, this method has limitations, such as the absence of a standardized protocol and a lack of both reproducibility and sensitivity. Considering these limitations, several works aimed to apply more standardized approaches to studying CF clinical isolates.

Boudet et al. used both the Biofilm Ring Test^®^ (BRT^®^) (BioFilm Control, Saint-Beauzire, France) and the BioFlux^TM^ 200 to study biofilm formation in the presence or absence of antibiotics by MRSA from CF patients [40]. BRT^®^ evaluates bacterial adhesion and early biofilm formation by immobilizing magnetic beads throughout the formation of biofilm. It is also dedicated to studying the capacity of antibiotics to inhibit biofilm formation through an approach called the Antibiofilmogram^®^ [45] whereas with the microfluidics system BioFlux™ 200, biofilm formation can be studied under dynamic conditions [46]. Both approaches were adapted for use with the mucin-containing synthetic growth medium, ASM [47,48], particularly the ASM had to be a modified (0.22 μm filtration) for use with the BRT^®^ which did not accept opaque media. They showed that adhesion was enhanced in ASM compared with BHI medium. They also brought new insights into CF MRSA’s ability to form biofilm, previoulsy little studied, showing that biofilm formation was strain-dependent, even for clonally related strains isolated from a same sputum sample, and differentially influenced by antibiotics. The importance of the patient’s colonization history was also highlighted as two MRSA strains, isolated three years apart in a chronically colonized patient, showed distinct patterns of biofilm formation (increased biofilm formation for the latest isolated strain) in the BioFlux™ 200 assay. More recently, Cheng et al., considering that in vivo biofilm may be formed in the absence of any surface for attachment, developed a surface-independent method to study biofilm formation based on a hanging-drop biofilm culture model with the aim of reducing the gap between in vitro predictions and in vivo responses [4]. The method appeared highly attractive for studying CF clinical isolates as, in CF, biofilm consists of unattached aggregates dispersed in the mucus [49]. Using this original, 96-well plate hanging-drop technology coupled with gene expression quantification and CLSM with differential fluorescent staining of biofilm components, the authors showed that the biofilm formed by a CF MRSA isolate displayed specific characteristics compared with that of an MRSA strain from a central catheter-related infection [4]. Indeed, this biofilm was less rich in matrix and had a lower viable cell count but was formed with double-sized micro-colonies and had a 50% higher metabolic activity. Studying the expression of three representative genes that are known to be upregulated in surface-attached *S. aureus* biofilms: *sdrC* (Ser-Asp-Arg-rich fibrinogen-binding protein) encoding proteins for fibrinogen mediated cell adhesion, *arcB* (ornithine transcarbamylase) involved in extraction and catabolism of arginine, and *ureC* (urease accessory protein C) involved in metabolism of urea, showed that these three genes were differentially upregulated over time in hanging-drop *S. aureus* biofilms compared with surface-attached ones supporting that hanging-drop biofilm maturation occurred earlier than that of surface-attached biofilm. In addition, *sdrC* expression levels were unchanged in hanging-drop model but dramatically upregulated in surface-attached biofilm model suggesting that the biofilm formation in the hanging-drop formation may not depend on fibrinogen-mediated adhesion [4]. Again, these observations highlighted the importance of using clinical isolates and testing methods that mimic CF lung conditions as closely as possible.

### 3.2. Ex Vivo Models

Using both immortalized human CF airway epithelial cells and primary CF human bronchial epithelial cells (obtained from the explanted lungs of CF patients), Kiedrowski et al. quantified biofilm biomass produced by *S. aureus* during co-infection with Respiratory Syncytial Virus (RSV) in live-cell imaging chambers. They observed that *S. aureus* biofilm growth was enhanced when cells were coinfected with RSV and that factors secreted during viral infection benefitted *S. aureus* biofilms. This observation obtained with RSV and *S. aureus* reference strains was also observed with human Rhinovirus 14 and confirmed with CF chronic rhinosinusitis clinical isolates [19]. Two other publications from the same team studied *S. aureus* biofilm formation after histological staining in an ex vivo model of CF infection comprising pig bronchiolar tissue (ex vivo pig lung, EVPL) and the synthetic mucus SCFM in the presence or absence of antibiotics [50,51]. They confirmed that a greater proportion of *S. aureus* localized as aggregates in the mucus (unattached biofilm) rather than associated with tissue. Sweeney et al. also provided interesting comparative results for (i) a pair of *S. aureus* strains isolated during lung exacerbation and stable clinical status showing differences in both growth and the location of bacterial cells and (ii) three lungs showing that growth of the strains depended on the lung tissue inoculated and thus on the host [50]. Regarding the effects of antibiotic treatment on biofilm formed on EVPL, clinical *S. aureus* isolates displayed an increased tolerance to antibiotics (linezolid, flucloxacillin) with effects not correlated with the Minimum Inhibitory Concentration (MIC). Thus, the EVPL model was reported as a host-mimicking model suitable for accurate antimicrobial susceptibility testing of CF pathogens [50,51].

All these methodological development efforts underline the challenge of studying biofilm formation in the context of CF and it probably remains illusory to mimic all the environmental stresses and conditions found in CF airways (acidity, anaerobiosis, inflammation and antimicrobial conditions—peptides, antibiotics; etc.). They also underline the different behavior of *S. aureus* strains according to the patient’s colonization history (because during chronic colonization, adapted strains are isolated) or clinical status (stable or with pulmonary exacerbation). This should be borne in mind when analyzing the literature and trying to compare results from different studies.

## 4. Biofilm Formation by *S. aureus* in the Unique Context of Cystic Fibrosis

### 4.1. Observational Studies

The studies reviewed in this part of the manuscript are summarized in Table 1 and complementary data are given in Supplementary Table S1. When detailed, strains forming biofilm are usually classified into different categories: non biofilm-producers, weak/minor, moderate/medium/intermediate and strong biofilm-producers. However, different criteria for biofilm production classification may be used according to the study. Unless otherwise specified, the studies investigated biofilm biomass formed by CF *S. aureus* strains through indirect measurement via CV staining.

#### 4.1.1. Overall Ability of *S. aureus* to Form Biofilm in CF

First of all, we addressed the question of whether CF strains may have specific capacities for biofilm formation. We found two observational studies that compared the biofilm-forming ability of *S. aureus* from CF and non-CF patients with divergent results. In the study by Molina et al., comparing 17 strains collected from CF respiratory samples and 20 strains from blood cultures of non-CF patients, nearly all strains formed biofilm and no significant difference in biofilm formation between strain categories was observed (16 out of the CF strains, 94.1%, versus all the non-CF strains were biofilm-formers) [52]. By contrast, the study by Cakir Aktas et al. showed that biofilm production was significantly more often observed among 31 CF methicillin-susceptible *S. aureus* (MSSA) compared with 57 non-CF *S. aureus* isolates collected from hospitalized patients with lower respiratory tract infection (53.4% MRSA, 46.6% MSSA). Biofilm production was indeed detected in 96.8% of CF strains versus 47.4% of non-CF strains, suggesting that *S. aureus* may have a particular ability to form biofilm in the context of CF [53].

We then examined other studies reporting the overall proportion of CF strains displaying any ability to form biofilm. Taken together, three studies using the same classification criteria [54] examined a total of 86 CF *S. aureus* strains including 16 MRSA and 70 MSSA. Biofilm production was detected in 74.4% of the strains distributed in 25.6% of weak, 41.9% moderate and 7% strong biofilm-producers [35,55,56]. Other studies each applied specific criteria for strain classification according to their biofilm formation. Among the strains forming biofilm in the Cakir Aktas et al. study (96.8% of 31 MSSA), 32.3% exhibited strong, 38.7% moderate and 25.8% weak positive phenotype [53]. Wieneke et al. showed that most *S. aureus* isolates were minor biofilm-forming strains (including non-biofilm-producers, weak and moderate biofilm-producers in that study) (1773/2319 isolates, 76.5%), while 546 isolates (23.5%) were strong biofilm-producers [57]. Biofilm formation was detected for all the 14 CF MRSA included in another study with 71.4% of strains being strong biofilm-producers [58] and in 80% of the *S. aureus* strains examined by Pompilio et al. [59]. Finally, using the BRT^®^ and ASM, 55.6% of 63 CF MRSA were shown to form early biofilm and were classified according to the biofilm formation index value generated by the device in 28.6% of intermediate and 27% of strong biofilm-producers [40].

We finally examined studies evaluating biofilm formation over time during chronic colonization of CF airways by comparing biofilm formation of *S. aureus* strains collected early in the course of CF airway infection (early strains) and long-term adapted *S. aureus* strains (late strains). The seven studies retrieved showed different results, as four studies including few strains found a positive association between biofilm production and the persistence of *S. aureus* over time in CF patients [40,60,61,62] whereas, for studies including a larger number of *S. aureus* strains, one reported no change [63] and two others highlighted the variable evolution of biofilm formation over time according to the patient [57,64]. These studies are presented successively hereafter and in Table 1.

**Table 1:** Summarized presentation of studies on biofilm formation by clinical strains of *S. aureus* isolated from patients with Cystic Fibrosis.

CF Patients		*S. aureus* Strains		Biofilm Formation	Ref.
n	Country	n (MSSA/MRSA)	CF Host-Adapted Strains	Biofilm-Producing Strains (%) & Evolution Weak (W)/Moderate (M)/Strong-Producers (S) (%)	
18	Spain	93 (0/93)	Persistence of a single MRSA clone: 77.8% of patients	14/15 CF MRSA pulsotypes	[52]
31	Turkey	31 (31/0)	NA	96.8% W (25.8%)/M (38.7%)/S (32.3%)	[53]
183	Iran	24 (20/4)	NA	66.6% W (37.5%)/% (20.8%)/S (8.3%)	[55]
NA	Poland	33 (30/3)	NA	90.9% W (24.2%)/M (60.6%)/S (6.1%)	[35]
15	Italy	15 (8/7))	NA	80%	[59]
42	Italy	SCV: 28 (21/7) Non-SCV: 29 (20/9)	Patients chronically colonized	SCV positive strains: 100% W (25%)/M (53.6%)/S (21.4%) Non-SCV (normal phenotype) strains: 62% W (17.2%)/M (37.9%)/S (6.9%)	[56]
14	Germany	2319 (unk.) 501 mucoid	Mean persistence: 15.6 y (range: 10–21 y)	No + W + M (76.5%)/S (23.5%) Evolution: unchanged: 8/:4/:2 patients	[57]
5	Italy	14 (0/14)	Persistance: 5; chronic colonization: 2 patients	100% (M and S)	[58]
35	France	63 (0/63)	Chronic colonization: 16/35	No (44.4%)/M (28.6%)/S (27%)	[40]
2	France	2 (unk.)	Chronic colonization: 1 patient	100% Late isolate: 5 x more biofilm than early isolate (from another patient)	[60]
3	France	6 (2/4) 3 early, 3 late	Late isolates: 3/6 (interval early/late: 2.8–9 y)	100% More biofilm formed by late isolates	[61]
9	Germany	18 (6/12)	Late isolates: 9/18 (interval early/late: 3–13 y)	11.1% (although all carried *icaA*, *C* and *D* genes)	[62]
49	U.S.A.	98 (0/98)	Late isolates: 49/98 (interval early/late: ≥ 2 y)	100% No differences between incident/chronic isolates	[63]
29	Germany	58 (56/2)	Mean persistence: 8.25 y (range: 5.1–13.6 y)	W: 66% Evolution: unchanged: 17/:7/:5 patients	[64]
8	Germany	425 (unk.) 115 mucoid (all carried the 5 bp-deletion)	Mean persistence of 29 m (range: 1–126 m)	All mucoid isolates: enhanced biofilm production, Non-mucoid strains: almost no biofilm formation	[65]
81	Germany	1050 (unk.) 37 mucoid (25 carried the 5 bp-deletion)	NA	6/7 patients: mucoid isolates formed significant higher amounts of biofilm than non-mucoid isolates; 1/7 patients: no biofilm formed by *Sa* with mucoid phenotype (no 5 bp-deletion)	[66]
NA	U.S.A.	50 (unk.)	NA	86% (including CFSa36 strain, see text)	[67]
2	Ireland	2 (unk.)	NA	100% when stimulated by bile or bile acids	[68]
3	NA	12 (0/12)	12/12	100%	[69]

MSSA, methicillin-susceptible *Staphylococcus aureus* (*Sa*); MRSA, methicillin-resistant *S. aureus*; SCV, small colony variant; unk., unknown; y, years; m, months; NA, not available; No: non-biofilm formers.

Ciornei et al. compared two *S. aureus* clinical isolates, one isolated at the beginning of infection and one at a chronic stage in another patient. Despite the limitation that the two strains were not isolated from the same patient, the late isolate produced almost five times more biofilm than the early isolate with the continuous-flow culture bioreactors [60]. Congruent observations were made for strains successively isolated in the same patient. Two paired MRSA strains isolated three years apart from the sputum of the same CF patient showed that the late strain systematically displayed higher biofilm formation than the early strain using the BioFlux^TM^ 200 system, whatever the experimental condition (BHI medium or ASM) and measured time point (5, 12, 24 or 36 h) [40]. Similarly, Tan et al. investigating one pair of MSSA and two pairs of MRSA demonstrated that late isolates had greater ability to form biofilm than early paired isolates, with a mean time between the collection of early and late isolates estimated at 6.2 years [61]. Of the nine early/late strain pairs investigated by Treffon et al., two of the late isolates became biofilm-producers, one livestock-associated MRSA and one MSSA, after eight and 13 years of colonization, respectively, whereas all other strains were non-biofilm formers [62]. By studying a larger number of *S. aureus* strains (49 pairs of CF MRSA), Gilpin et al. did not observe any significant differences in biofilm production between early and late isolates collected at least two years apart, although all strains showed the capacity to form biofilm [63]. Finally, two studies showed that *S. aureus* persistence was associated with variable evolution in biofilm formation. In a one-year observational prospective study, Wieneke et al. examined a total of 2319 CF *S. aureus* strains (methicillin-resistance not specified) collected from the sputum of 14 CF patients with long-term persistent infections (range: 10–21 years of persistence) [57]. They observed that biofilm formation had increased in four (29%), was unchanged with a large percentage of high biofilm-forming isolates in three (21%), unchanged with no biofilm-positive isolates in five (36%), and had decreased in two (14%) patients over time. Hirschhausen et al. investigated 29 pairs of *S. aureus* (including 3.4% MRSA and 96.6% MSSA) collected from the sputum of CF patients with a mean persistence of 8.25 years (range: 5.1–13.6 years) [64]. Biofilm formation had increased in seven (24%), was unchanged in 17 (59%) and had decreased in five (17%) late isolates compared with early isolates.

The variability observed in biofilm production both between and within studies might be explained by numerous confounding factors like the length time of observations and also the timeline of strain isolation regarding the patient’s colonization history (early strain are not necessarily isolated during the initial or first episodes of infection). The influence of co-infecting pathogens such as *P. aeruginosa* is also a major factor of variability of *S. aureus* biofilm formation (see also part 6) [57]. Finally, Hirschhausen et al. hypothesized that it might be more appropriate for *S. aureus* survival to increase biofilm formation at the onset of chronic colonization to protect itself against phagocytosis and antibiotic treatment whereas, during long-term persistence, it might be more suitable to form less biofilm to be able to disseminate in the airways of CF patients [64].

Altogether, biofilm formation was a common trait of both MRSA and MSSA strains in CF patients ranging from 55.6% to 100% of the strains studied according to the study with a proportion of strong biofilm-producing strains varying from 7 to 71.4%. As indicated, such variability may be associated with distinct methods of biofilm formation study or criteria applied for interpretation, to distinct studied strains and patient colonization history (MRSA vs. MSSA, strains from early episodes of infection vs. adapted strains from chronically colonized patients, strains from patients co-colonized by other pathogens or not). The clinical impact of biofilm formation has yet to be investigated but Wieneke et al. showed that high biofilm-forming *S. aureus* isolates were associated with fewer pulmonary exacerbations in CF patients and, conversely, that exacerbations had a negative impact on biofilm formation [57].

#### 4.1.2. Biofilm Formation in Specific Subpopulations of CF *S. aureus* Strains

To date, we only found two studies that have compared MRSA and MSSA in their ability to form biofilm but only one included CF strains. In the latter study, Pompilio et al. showed a significantly higher median biofilm amount produced by MRSA compared with MSSA but this study was based on a few CF strains (three MRSA and three MSSA) [70]. These results were congruent with those obtained by Kadkhoda et al. on *S. aureus* collected from children with clinical symptoms of infection admitted to Children’s Medical Center Hospital in Tehran showing an association between biofilm formation and MRSA [71]. It thus remains interesting to study this association on a larger number of CF strains, particularly because studies suggested that mechanisms governing biofilm formation might be distinct in MRSA compared with MSSA. Indeed, intercellular adherence (*ica*) operon was shown to mediate biofilm formation in MSSA through the production of polysaccharide intercellular adhesin (PIA), also known as poly-N-acetyl-β-(1-6)-glucosamine (PNAG) whereas an *ica*-independent mechanism involving the fibronectin binding proteins, FnBPA and FnBPB, and the major autolysin Atl appears to play an important role in MRSA biofilm development [72,73].

Regarding small colony variants (SCV), which are slow-growing auxotrophic subpopulations of bacteria that play an important role in persistence in the CF lung, Morelli et al. observed that 28 SCV strains (7 MRSA and 21 MSSA collected from CF patients) showed a significantly higher ability to form biofilm than 29 strains with normal phenotypes (100% versus 62%) but also comprised a higher proportion of strong biofilm-producers (21.4% versus 6.9%) [56]. In that study, strong hypermutators also showed a greater ability to form biofilm than non-mutating strains, although the difference observed did not reach statistical significance.

Finally, an unusual mucoid phenotype was described for *S. aureus* [74] and further identified in 2.5% (8/313), 8.6% (7/81) and up to 71% (10/14) of CF patients with positive *S. aureus* cultures, either MRSA or MSSA [57,65,66]. The higher prevalence of mucoid isolates in the study by Wieneke et al. [57] compared to the two earlier studies [65,66] might be explained by the lower number of patients included, the patient selection criteria implying a long period of *S. aureus* persistence (mean duration of 15.6 years) during which the number of SCVs increased longitudinally and the deep culturing conditions. Nevertheless, congruent results were observed in all three studies showing that mucoid *S. aureus* strains produced significantly higher amounts of biofilm compared to non-mucoid strains [57,65,66]. Conversely biofilm formation was also associated with the mucoid phenotype [57]. Mucoidy associated with high biofilm formation thus represents survival advantages in the CF lung thereby certainly contributing to the prolonged persistence of *S. aureus* in patients’ airways.

### 4.2. Factors Influencing Biofilm Formation in CF and the Molecular Mechanisms Governing It

*S. aureus* biofilm formation, as well as the genes and proteins involved in biofilm production have been widely described in contexts other than CF [3]: FnBPA and FnBPB [72,75], *Sa* G5-E repeat protein SasG, the Clf-Sdr family consisting of clumping factor A/B (ClfA/B) and the serine-aspartate repeat family (Sdr) proteins, *ica* operon (*ica*ADBC) transcription resulting in the expression of PIA/PNAG. Similarly, different regulators were characterized such as the intracellular adhesin locus regulator (*ica*R), the teicoplanin-associated locus regulator (*tcaR*), the staphylococcal accessory regulator (*sarA*) and the accessory gene regulator (*agr*) [67,76,77,78] (for a recent review, see Schilcher & Horswill [79]).

In the following subsections, we describe the different factors that were shown to influence biofilm formation of *S. aureus* strains from CF patients, i.e., host response, environmental factors (metal ions, anaerobic conditions, bile acids), mutations and altered gene expression, and mucoidy, as well as the genes and molecular mechanisms that are critical for biofilm formation by *S. aureus* in the unique context of CF. These factors remain poorly explored in the literature and are promising targets for fighting persistent *S. aureus* infections especially in patients with CF.

#### 4.2.1. Biofilm Formation and Host Response

Sadowska et al. studied two CF *S. aureus* strains (methicillin resistance not specified) trying to understand “frustrated phagocytosis”, i.e., the weak activity of phagocytic cells against microbial biofilm [80]. They showed that *S. aureus* cell wall components, such as peptidoglycan and lipoteichoic acids, could influence the stimulation of leukocytes resulting in various amounts of cytokine production, including interleukin (IL)-10, IL-6 and tumor necrosis factor-α. However, although these immunomodulatory properties were observed for biofilm-forming CF *S. aureus* strains, they were not specific to these strains and also present in their planktonic counterparts, as already shown by Ciornei et al. [60].

#### 4.2.2. Biofilm Formation and Environmental Factors

Biofilm formation by *S. aureus* in CF is affected by environmental factors including metal ions. Indeed, in an MRSA strain called CFSa36 (sequence-type not determined), Liu et al. identified a putative cobalt transporter ATP binding domain (CbiO) that was required for biofilm formation. They showed that the copper ions (Cu^2+^) entirely complemented the capacity of the *cbiO* knockout mutant to form biofilm in a dose-dependent manner without having any impact on bacterial growth. Conversely, iron ions (Fe^3+^) significantly decreased the ability of MRSA to form biofilms in a dose-dependent manner. Thus, they hypothesized that CbiO might mediate *S. aureus* biofilm formation by affecting the transport of copper ions [67,81].

Anaerobic conditions, typically observed in the CF lung due to mucus obstruction, can also influence *S. aureus* biofilm production. By studying 98 CF MRSA, Gilpin et al. showed that biofilm formation was significantly lower under anaerobic conditions than under aerobic conditions [63]. It should be noted that this finding on clinical MRSA strains differed from that reported by Cramton et al. and Ulrich et al. on reference MSSA strains, who demonstrated that anaerobic in vitro growth conditions triggered increased *ica* gene transcription and PIA/PNAG expression by *S. aureus* and consequently, biofilm formation [82,83]. Because of these opposite observations between studies including strains of distinct origins and methicillin-susceptibility, additional investigations are still needed to decipher the influence of anaerobic conditions on biofilm formation by *S. aureus*.

Bile acids are another environmental factors affecting biofilm formation. On two *S. aureus* strains from CF children (specimen collection site and methicillin resistance not specified), Ulluwishewa et al. demonstrated that physiologically relevant concentrations of bile and, more precisely, sodium cholate and sodium deoxycholate bile acids enhanced biofilm formation [68]. Regulatory mutations involved in cell wall teichoic acid synthesis (surface-exposed anionic glycopolymers bound to the peptidoglycan layer) apparently induce greater sensitivity to bile causing increased cell wall stress. Thus, in the presence of sub-inhibitory concentrations of bile, *S. aureus* develops an adaptive response by increasing biofilm production. Although bile-stimulated biofilm formation was also observed in two community-acquired MRSA strains from non-CF patients, suggesting that it is not a CF-specific phenomenon, it may be more prevalent in CF patients as up to 40% of CF children and 80% of CF adults can suffer from gastro-oesophageal reflux disease resulting in the presence of bile acids into the lungs [68,84].

#### 4.2.3. Biofilm Formation, Mutations and Altered Gene Expression

Tan et al. compared the genomes of paired early and late *S. aureus* strains (one pair of MSSA and two pairs of MRSA) collected from chronically infected CF patients. Mutations and altered gene expression occurred over time, some of which seems to give CF *S. aureus* strains greater ability to produce biofilm, i.e., mutations in the *fakA* (fatty acid kinase A) gene and mutations in the *saeR* (*Staphylococcus* exoprotein expression protein R) gene causing an up-regulation of SdrD adhesin involved in biofilm production, down-regulation of the *agr* regulon resulting in an overexpression of adhesins [61]. Indeed, apart from the *fakA* gene currently little described in *S. aureus*, these genes have already been reported as being involved in *S. aureus* biofilm production [69,85,86]. Gabryszewski et al. documented genomic and transcriptional changes in some, but not all, CF host-adapted MRSA strains. First, they observed that mutations and altered expression of *dacA* and *gpdP* genes correlated with increased biofilm formation over time. The *dacA* gene encodes a deadenylate cyclase required for the synthesis of cyclic-di-AMP (c-di-AMP), a multifunctional secondary metabolite involved in biofilm formation whereas *gpdP* encodes a phosphodiesterase responsible for c-di-AMP hydrolysis [69]. These mutations had already been shown to affect *Sa* biofilm formation by Corrigan et al., and are not specific to CF strains [87]. Moreover, as also described by Tan et al., they observed mutations in the *saeR* gene [61,69]. However, Gabryszewski et al. also found changes in the expression of certain genes, so far little described in the literature, which may be specific to biofilm formation by *S. aureus* in CF patients: increased expression of specific metabolic genes including *gapR* (which encodes a central glycolytic regulator), *zwf* (involved in the pentose phosphate pathway) and oxidative tricarboxylic acid (TCA) cycle genes like *sdh* and *mqo1* as well as decreased expression of *gltA*, all of which lead to biofilm production [69]. Interestingly, in all CF host-adapted MRSA isolates, a 10,000-fold increase was observed in the *fumC* gene expression that functions primarily as a malate dehydratase increasing fumarate concentrations not significantly assimilated by MRSA strains. Thus, in CF patients, MRSA strains undergo metabolic reprogramming with minimal consumption of TCA cycle substrates (which limit pro-oxidant generation) and conversely increased assimilation of pyruvate and glucose polymers which promote the production of extracellular polysaccharide-composing biofilm, such as PIA/PNAG (inherently anti-oxidant and acting as an intercellular adhesin [4]). By protecting bacteria from oxidative stress and promoting biofilm formation, all these mechanisms allow MRSA isolates to persist within the CF lung and develop chronic infections [69].

#### 4.2.4. Biofilm Formation and Mucoidy

Schwartbeck et al. not only showed that mucoid *S. aureus* strains isolated from CF patients were hyper-producers of PIA-associated biofilms but also described the underlying molecular mechanism for mucoidy and its impact on biofilm formation [65]. The 115 mucoid strains investigated displayed a diversity of genetic background (Sequence type (ST)25, ST188, ST5, ST30, ST7 and ST1909) but all of them carried the same 5 bp-deletion (TATTT), in the same intergenic region between *icaR* and *icaA*. Deletion affected binding of the repressor of biofilm “rob” [88], resulting in increased expression of PIA/PNAG. In addition, in two CF patients persistently infected by mucoid *S. aureus* strains, Schwartbeck et al. identified non-mucoid and non-biofilm-forming late isolates without PIA/PNAG hyperexpression, and carrying the 5 bp-deletion. They demonstrated that various compensatory mutations in *icaA*, *icaD* and *icaC* caused the abrogation of biofilm formation and a non-mucoid phenotype. Thanks to whole genome sequencing, they confirmed that mucoid isolates evolved from the non-mucoid *S. aureus* clonal strain and that the isolates with both 5 bp-deletion and compensatory mutations evolved from the mucoid isolates. For some, but not all mucoid isolates, mucoidy might thus confer a short-term advantage but, due to mucoidy-associated fitness loss, not a long-term advantage for persistence, resulting in the emergence of compensatory mutations. This corroborated certain observations of a decrease in biofilm formation over time and the hypothesis drawn by Hirschhausen et al. that, after an initial increase in biofilm formation to enhance protection during establishment in the CF lungs, decreasing biofilm formation could be more appropriate for dissemination and persistence [64].

#### 4.2.5. Biofilm Formation and Bacterial Interactions

Pulmonary infections in CF patients are considered as polymicrobial [89,90,91] and studying a single pathogen has limitations with results that probably do not reflect the bacterial behavior of the CF lung. With the diversity of bacterial species and abundance of the community present in the airways of CF patients, interactions between bacteria and competition for space and nutrients can indeed be established through physical proximity and chemical communication pathways [92,93]. The close relationships within multi-species biofilms facilitate these bacterial interactions and examining the interspecies interactions affecting biofilm formation and multi-species biofilms is certainly more representative of the conditions in the CF lungs, although it is probably impossible to fully grasp the complexity of this environment through laboratory experiments [94,95,96,97].

Diverse associations of pathogens may be observed in CF patients [98], the most studied of which being the *S. aureus*-*P. aeruginosa* as this is the co-infection most frequently observed in CF patients. Although co-infections with *Haemophilus influenzae* are observed before adulthood (less than 10% of patients colonized by *Sa*), the majority of co-infections are indeed those observed with *P. aeruginosa*. A study on 134 patients from 2004 to 2017 managed to identify *S. aureus*-*P. aeruginosa* co-infections in 30–50% of patients [99]. More generally, co-infection by these two species has been identified in 28.3% of CF patients (8 studies and 1432 patients) [100]. A similar observation was made as far as dual-species biofilms are concerned, *S. aureus* being mostly studied in association with *P. aeruginosa*. Nevertheless, among all these studies, we noted that (i) most studies on the *P. aeruginosa*-*S. aureus* combination focused on *P. aeruginosa* with few or no results on *S. aureus* [101] and (ii) *P. aeruginosa* co-studied strains were mostly reference strains, i.e., PA14, originating from a wound on a burns patient [102,103], PAO1, a moderately virulent strain isolated from a wound over 50 years ago [22,39] and strain ATCC 27,853 from blood culture [96]. As in other parts of this review, we voluntarily focus hereafter on data obtained from CF *S. aureus* and *P. aeruginosa* clinical strains, with the aim of reporting results as close as possible to the specific clinical context of CF. A single exception was made for the publication by Barraza & Whiteley that studied laboratory strains of *P. aeruginosa* (PA14) and *S. aureus* (LAC) but used synthetic CF sputum medium (SCFM2), to be as close as possible to CF lung conditions [39]. Regarding *S. aureus*-*P. aeruginosa* studies, we noted a major lack of studies on clinical strains from CF patients as the majority of studies—even the most recently published ones—only included reference strains. The most relevant study among all those we selected is probably the one by Fugère et al. which included *S. aureus* and *P. aeruginosa* clinical strains co-isolated from the same patient. It showed that some results were specifically obtained when studying these pairs of strains, hence the need for such studies which are extremely rare in the literature [104].

First, regarding the overall architecture of biofilm, results obtained for laboratory strains in SCFM2 highlighted a modification in the structural frame of biofilm formed by *S. aureus* co-cultured with *P. aeruginosa* with (i) fewer *S. aureus* aggregates, *S. aureus* planktonic cells being more numerous, and (ii) *S. aureus* aggregates of smaller size [39].

**Figure 2:**
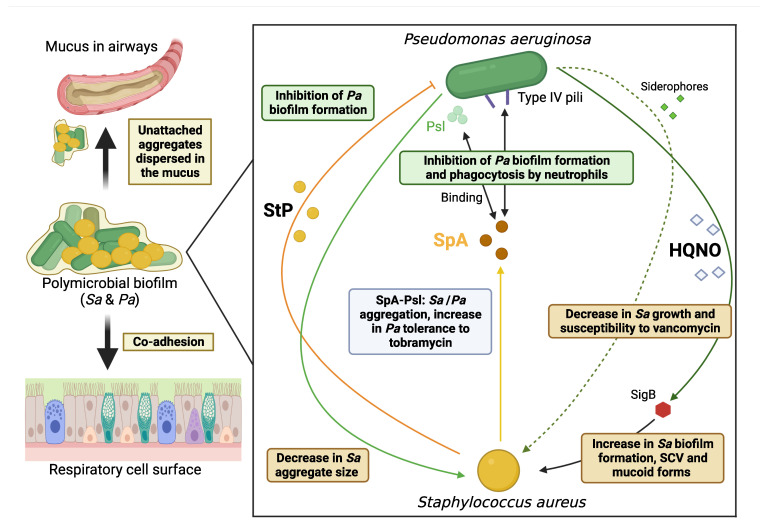
Diagram showing the major mechanisms involved in the interaction between CF *Staphylococcus aureus* (*Sa*, in orange) and *Pseudomonas aeruginosa* (*Pa*, in green) strains in dual-species biofilm. The text boxes specify the role associated with each molecule/mechanism. StP: Staphylopine; SpA: Staphylococcal protein A; HQNO: 2-heptyl-4-hydroxyquinoline N-oxide; Psl: Polysaccharide synthesis locus; SigB: Sigma Factor B; SVC: Small Colony Variant (figure created with Biorender.com (accessed on 31 October 2022)).

On a molecular level, *S. aureus* produces various exoproducts that can be sensed by *P. aeruginosa* [101,105]. Those linked to modifications in biofilm formation were: staphylopine (StP), a broad-spectrum metallophore with a central role in metal acquisition and *S. aureus* virulence, and the Staphylococcal protein A of *S. aureus* (SpA), a secreted virulence factor that affects host immune response and inhibits phagocytosis (Figure 2). Both exoproducts significantly decreased the biofilm biomass formed by clinical CF *P. aeruginosa* isolates. StP was shown to inhibit biofilm formation by *P. aeruginosa* by reducing the availability of zinc [101]. Interestingly, the study included strains co-isolated from three different CF patients showing that the interaction was consistently observed despite strain-to-strain variability. These observations were congruent with those of Menetrey et al. who tested *S. aureus* (chronic colonization) and *P. aeruginosa* (sporadic colonization) from the same CF patient via the CV-based method and showed a decrease in *P. aeruginosa* biofilm formation in *S. aureus*-*P. aeruginosa* co-culture compared with *P. aeruginosa* monoculture [106]. So far, this recently published observation on the implication of StP in the *S. aureus*-*P. aeruginosa* interplay has only been reported for strains originating from CF patients. On the other hand, SpA interacts with two important determinants of *P. aeruginosa* biofilm formation: i) exopolysaccharide, *encoded by the p*olysaccharide *s*ynthesis *l*ocus (*psl*), which is the predominant polysaccharide of the extracellular matrix of *P. aeruginosa* biofilm and a crucial component for its formation and ii) PilA, a component of the type IV pili of *P. aeruginosa* [107]. SpA was also shown to lead to bacterial aggregation in *P. aeruginosa* biofilms and the interaction between SpA and Psl was associated with an increase of *P. aeruginosa* tolerance towards tobramycin, an antibiotic commonly used against this pathogen in aerosol therapy [108].

On the *S. aureus* side, interacting with *P. aeruginosa* led to several modifications in characteristics, presented hereafter, all favoring the persistence of clinical CF *S. aureus* isolates (Figure 2).

Several studies have observed a correlation between HQNO (2-heptyl-4-hydroxyquinoline N-oxide) molecules of *P. aeruginosa* whose production is under the control of the *Pseudomonas* Quinolone Signal (PQS) quorum sensing system of *P. aeruginosa* and increased biofilm formation by *S. aureus* strains [104,109]. The role of these molecules was confirmed with supernatants from *P. aeruginosa* isogenic mutants deficient in PQS and HQNO production which significantly stimulated less biofilm formation by *S. aureus* [104]. Adding *P. aeruginosa* supernatants to *S. aureus* biofilms grown either on epithelial cells or on plastic also significantly decreased the susceptibility of clinical CF *S. aureus* strains to vancomycin [110]. HQNO molecules were again shown to be involved in this phenomenon [110], just as the two *P. aeruginosa* siderophores, pyoverdine and pyochelin, which also induce a decrease in *S. aureus* growth correlated with the decrease in susceptibility to certain antibiotics observed [109,110]. The observations made by studying *S. aureus* and *P. aeruginosa* strains co-isolated from the same CF patient were of great interest, showing that results vary depending on the origin of the strains, with less HQNO production by *P. aeruginosa* and a less stimulated *S. aureus* biofilm formation for co-isolated strains. These results highlighted the within-host co-evolution of *S. aureus* and *P. aeruginosa* adapting to the specific abiotic conditions of CF lungs and also to other pathogens present in the niche [104].

HQNO molecules also induce SCVs of *S. aureus*, a form adapted to intracellular survival [109]. Interestingly, CF patients with SCVs of *S. aureus* were significantly more frequently co-colonized with *P. aeruginosa* than patients colonized by *S. aureus* of normal phenotype (75% vs. 37.9%) [56]. Both the stimulation of biofilm production and the switch to SCV under HQNO exposure were shown to be dependent on the activity of the global *S. aureus* regulator Sigma factor B (SigB), a crucial factor for adaptation in chronic infections, which was previously linked to an increased expression of both the FnBPA-encoding gene and the biofilm-associated *sarA* gene [109,111]. Additionally, co-infection with *P. aeruginosa* selected for mucoid phenotype of *S. aureus* that was associated with high biofilm formation and this represented another adaptive characteristic of *S. aureus* for protectin and survival against attacks by small molecules produced by *P. aeruginosa* such as HQNO [57].

Finally, in the dual-species biofilm with *P. aeruginosa*, *S. aureus* may also undergo a switch to a Viable But-Non-Cultivable (VBNC) state that favors its survival and persistence in the co-colonized host. Molecular investigations revealed that several *S. aureus* genes involved in virulence (Quorum Sensing genes *sarA* and *hld*, biofilm formation gene *icaA*, cytotoxicity gene *cplP*, and stress response genes *sodA* and *uspA*) were overexpressed during this switch [112], suggesting that the phenotypic switching to VBNC state might account for *S. aureus* pathogenicity and be involved in the clinical outcome of the co-infection [112].

Introducing a third partner add complexity to the interactions. We found only one study on three species—the study by Tavernier et al.—in which *S. aureus* was studied in mixed biofilm assays with *Streptococcus anginosus* in the presence or absence of *P. aeruginosa* in a mucin-containing medium [113]. The community composition was shown to influence the antimicrobial susceptibility of partners in such multispecies biofilms. Changes in antimicrobial susceptibility were shown to depend on the antibiotic, the species and the strain involved, but *S. aureus* secreted compound(s) protected sessile *S. anginosus* from antibiotic killing whereas the antibiotic killing of *P. aeruginosa* was not influenced by the presence of *S. aureus* or *S. anginosus* and more *S. aureus* cells were killed by antibiotic treatment when grown together with *S. anginosus* and *P. aeruginosa* [113].

Despite growing interest in characterizing species interactions, their potential implications in the progression of polymicrobial pathologies are still poorly understood [100,114]. However, co-infection with *S. aureus* and *P. aeruginosa* correlates with a decline in lung function and a higher number of exacerbations and intravenous antibiotic treatments compared to infection with *S. aureus* or *P. aeruginosa* alone [100]. Particularly, co-infection with *P. aeruginosa* and MRSA is associated with the most severe clinical pictures compared to co-infections with *P. aeruginosa* and MSSA [100]. Furthermore, in young children co-infected with *P. aeruginosa* and MRSA, an increase in markers of lower airway inflammation was observed [115]. These studies all showed that there is an important need to further understand polymicrobial interactions [116], particularly in the context of multi-species biofilm, which have an impact on patients’ health. Altogether, reviewing this part of literature revealed reciprocal, complex interactions between *S. aureus* and *P. aeruginosa* affecting biofilm formation during Cystic Fibrosis but also that CF clinical isolates may display distinctively different traits from reference laboratory strains, thereby supporting the need for additional studies including clinically documented *S. aureus* strains from CF patients. These observations also warrant the need for studies of multispecies biofilms formed by *S. aureus* and other clinically relevant species in CF, either bacterial species such as members of the *Burkholderia cepacia* complex or emerging pathogens *Stenotrophomonas maltophilia* and *Achromobacter* spp., or fungal species. Indeed, interactions highlighted between *S. aureus* and these species in planktonic cultures [106,117,118,119] or in dual-species biofilms in contexts other than CF [120] should be explored further in biofilm conditions using CF clinical strains.

## 5. Anti-Biofilm Strategies Targeting *S. aureus* in CF

Limiting biofilm formation has thus become an important tactic considered in the development of new antimicrobial strategies. Regarding *S. aureus* and CF, several compounds including natural ones and antibiotics were shown to display anti-biofilm activity, whether limiting the formation of biofilm or affecting preformed biofilm.

### 5.1. Antimicrobial Peptides and Proteins of the Innate Immune System

Antimicrobial peptides (AMPs) are natural products of the immune system of particular interest with their broad-spectrum antimicrobial activity due to their disruptive mode of action against bacterial membranes occurring after electrostatic interaction between AMPs and bacterial cells [121]. Three cationic α-helical AMPs, i.e., two cathelicidin-derived peptides of bovine origin (bovine myeloid antimicrobial peptide (BMAP)-27, BMAP-28) and an artificial peptide P19(9/B) were tested on 15 clinical strains from CF patients [59]. Biofilm assays were performed using polystyrene plates and CV staining under reduced oxygen concentration, at acidic pH and in SCFM with the aim of simulating CF lung conditions. BMAP-28 and P19(9/B) at sub-inhibitory concentrations (1/2x MIC) were the most active with a significant decrease in biofilm formation (at least 25%) observed for 70% and 60% of the tested strains, respectively. At 1/4x MIC, BMAP-28 was significantly more active than the other two AMPs, still showing significant biofilm reduction in 50% of the strains compared to non-exposed controls. However, in all conditions, AMPs were shown to be less active than tobramycin in limiting biofilm formation. Testing combinations of AMP and tobramycin, the three AMPs showed either a synergistic effect with tobramycin or indifference to it [59].

More recently, Japonicin-2LF, an AMP secreted by the skin of the amphibian *Limnonectes fujianensis*, was shown to be particularly effective against two sessile MRSA CF strains through membrane permeabilization [122]. Interestingly, in vitro experiments showing biofilm disintegration under Japonicin-2LF challenge were completed by in vivo investigations showing that using this AMP was associated with a significant decrease in mortality of *Galleria mellonella* larvae infected by MRSA whereas no death of larvae were observed after injection of two doses of Japonicin-2LF. This AMP appeared to be a potential candidate for further evaluations as cytotoxicity, haemolytic activity and lactate deshydrogenase release were observed with high concentrations of 64 μM of Japonicin-2LF alone [122].

Human SPLUNC1 (short palate lung and nasal epithelial clone 1) is a protein of the innate immune system secreted in the human respiratory tract. It acts as a surfactant and regulates the epithelial sodium channel (ENaC) whose deregulation in CF worsens the mucus dehydration and ion imbalance due to a defect in CFTR [123]. Although human SPLUNC1 proteins were ineffective against biofilm formation by *S. aureus*, unlike the observations made for *P. aeruginosa* and *B. cepacia*, proteins modified by the addition of negatively charged residues in the α1−α4 region showed increased anti-biofilm activity against *S. aureus*. Despite the origin of the clinical *S. aureus* strains used in this study is unclear, the study showed the importance of this region of the protein in anti-biofilm activity against *S. aureus*. A further study addressed the antibiofilm activities of SPLUNC1-modified proteins and SPLUNC1-derived peptides against six MRSA strains isolated from CF patients [124]. A focus was made on modifications of the α4 helix that shared a similar structure with cationic AMPs and one peptide named α4M1 with enhanced amphipathicity was shown to reduce biofilm formation to 1 to 20% of the initial value after 24 h of incubation according to the strain tested. Cytotoxic evaluation did not show any hemolytic activity, even at high peptide concentrations up to 100μM allowing the pursuit of investigations on this peptide in a perspective of use in diverse biofilm-associated diseases.

### 5.2. Natural Compounds

Essential oils are complex mixtures derived from plants and may display certain antimicrobial properties. Papa et al. studied the anti-biofilm effect of 61 essential oils (EOs) on three CF clinical isolates 4S, 5S and 19S (including two MRSA and one MSSA) compared with two reference laboratory strains using microtiter plate biofilm assay and CV staining [125]. All EOs were tested for biofilm growth inhibition at 1.00% *v/v* concentration and it was found that several EOs had the ability to inhibit biofilm formation by one or several *S. aureus* CF strains. EOs from *Piper nigrum* (black pepper) (EO45) and *Mentha suaveolens* (sweet mint) (EO58) were selected for an in-depth study based on their constant activity on the studied *S. aureus* strains and biofilm reduction of up to 40% or more of its initial value, despite their different compositions. We may note that neither EO45 nor EO58 had any antibacterial activity on the CF clinical isolates showing an antibiofilm effect unrelated to the inhibition of bacterial growth. SEM analyses made it possible to visualize the highly disruptive action of both EOs on *S. aureus* biofilm showing a deconstructed surface and EPS disintegration. However, the dose-dependent effect of EOs was also highlighted as lower concentrations (0.05% v/v) mostly ended up abolishing the inhibition effect on biofilm formation and even enhanced biofilm growth in certain cases. Finally, chemical analysis led to the identification of the EO constituents related to the most effective biofilm growth inhibition as being eugenol, β-caryophyllene and, partially, β-pinene. Because of their various distinct components, essential oils probably have a multi-target action as each of the EO compounds may exhibit a different mechanism of action against biofilm formation by *S. aureus* strains from CF patients [125]. In a general manner, EOs have been shown to disrupt membrane integrity and metabolic pathways of the targeted pathogens [126]. Other phytochemicals, namely, combination of borneol and citral, and Pickering emulsions—stabilized by solid particles—of these compounds, were recently tested for anti-biofilm activity on the CF clinical isolate P8-AE1 in SCFM2. They showed inhibitory activity on biofilm formation and eradication of established biofilm (24-h-old *S. aureus* P8-AE1 biofilms) associated with G. mellonella larvae protection from *S. aureus*-induced killing [127]. Encapsulation increased their anti-biofilm activity while confering reduced toxicity and enhanced stability. Pickering emulsions thus represent attractive formulations to improve the efficacy of phytochemicals against biofilms.

Among the numerous complementary, alternative medicine practices, CF patients may use herbal therapy. In this context, the phenolic-rich fraction of an extract of the aerial parts of *Pulmonaria officinalis* was investigated for anti-adhesion and anti-biofilm properties on 20 clinical isolates from the sputum of children (1.5–19 years) with CF and chronic respiratory tract infection. The assay used inert polystyrene surfaces conditioned or not with mucin and elastin to mimic respiratory tract mucosa [42]. Adhesion was reduced by about 54% on the inert surface, 20–36% on mucin-coated surfaces and 14–45% on elastin-coated surfaces. Effects that were strain- and extract concentration-dependent without associated loss of *S. aureus* viability were suggestive of variable reduction in surface adhesin expression. However, subsequent observation of biofilm reduction did not reach significance and biofilm may even be increased under certain conditions; the extract was not effective in eradicating preformed biofilm either [42]. However, other observations like a significant reduction in α-toxin and sortase A (SrtA) activities warrant further exploration, as both α-toxin and SrtA were previously related to biofilm formation in other settings. SrtA is a transpeptidase involved in the cell wall anchoring of the MSCRAMMs. These surface-exposed molecules recognize extracellular host proteins such as fibrinogen and collagen, and are therefore essential in host-bacteria interactions, the first step of adhesion, biofilm formation and invasion. Reducing SrtA production will therefore limit bacterial adhesion to the host tissues leading to a decrease in biofilm production [128] making SrtA inhibitors promising anti-biofilm and anti-virulence compounds [129]. Alpha-toxin/alpha-hemolysin (HlA) production was also shown to be required for biofilm formation by *S. aureus* in several studies [130,131,132]. This transmembrane pore-forming multimeric toxin appeared required for cell-to-cell interactions during biofilm formation [130]. Others suggested that the lysis of underlying host cells by HlA may provide a nutrient source for *S. aureus* enhancing adhesion and inducing the production of biofilm components [132]. Again, anti-HlA compounds represent an alternative anti-*S. aureus* strategy as they might reduce not only epithelial toxicity but also biofilm formation on host tissues.

Secondary metabolites from lichens, more precisely, the fungal component of lichens, were first challenged for antibiofilm activity on CF *S. aureus* strains by Pompilio et al., 2013. Usnic acid and atranorin, which displayed antimicrobial activity on six *S. aureus* strains from CF patients (three MSSA, three MRSA), were evaluated for their activity against adhesiveness, biofilm formation and preformed biofilm using CV staining. Both secondary metabolites at 1/2x MIC affected adhesion of all strains and biofilm formation in all strains except one (unaffected by usnic acid only). Atranorin also decreased bacterial adhesion at lower concentrations (1/4x and 1/8x MIC) (75% reduction of adhesiveness compared with control for most conditions). At these subinhibitory concentrations, usnic acid and atranorin displayed variable results on biofilm formation depending on the *S. aureus* strain and metabolite concentration, with the highest activity for atranorin on MRSA and usnic acid on MSSA. Both metabolites were also significantly active on biofilm preformed by the two strongest biofilm-producing strains in this study (Sa3 and Sa15 MRSA) whatever the concentration tested (1x MIC and bactericidal concentrations 5x and 10x MIC) [70]. The effects of usnic acid on biofilm formation were further investigated on Sa3; ultrastructural and proteomic observations showed bacterial cell wall alterations and a decrease in the biosynthesis of amino acids and proteins essential to bacterial viability [133]. Regarding the anti-adhesion and anti-biofilm effects previously observed, usnic acid was shown to significantly reduce the transcription of the genes encoding the host matrix-binding proteins (elastin, laminin, fibronectin), as well as genes encoding lipase and thermonuclease. A dose-dependent effect was also observed on agrA expression. These modifications led to the inhibition of the first stage of biofilm formation (adhesion) which further contributes to biofilm formation reduction [133]. However, despite the clinical relevance of the anti-biofilm effects of usnic acid, its use is still under investigations as suitable formulations need to be developed due to toxicity issues [70].

### 5.3. Antibiotics

Despite antimicrobial resistance may be drastically increased in biofilm [7,50,51,134,135,136], antibiotics remain the therapeutic of choice for CF patients. Besides their antibacterial growth properties, antibiotics may also have an anti-biofilm effect. As seen in previous parts of this review, the anti-biofilm activities of antibiotics on clinical strains isolated from CF patients were mostly studied against *P. aeruginosa*. For *S. aureus*, an antibiofilm effect was observed with tobramycin, the comparator used to evaluate AMP’s anti-biofilm activity against *S. aureus* CF strains [59]. In this study, tobramycin showed the ability to significantly reduce biofilm formation (at least 25% for the three CF *S. aureus* strains tested), up to the lowest sub-inhibitory concentration evaluated (1/8x MIC). The Antibiofilmogram^®^ approach by BRT^®^ in filtered ASM was also used to study the effect of five antibiotics (trimethoprim, rifampicin, linezolid, ceftobiprole and ceftaroline) on biofilm formation by 17 strongly adherent/strong biofilm producers MRSA strains from CF patients [40]. Trimethoprim was totally ineffective at limiting adhesion and early biofilm formation, rifampicin was active on a highly limited number of strains (18%) whereas linezolid, ceftobiprole and ceftaroline were able to inhibit biofilm formation (biofilm MICs under the corresponding resistance threshold) of 65%, 70.5% and 76.5% of the strains, respectively. Biofilm formation by MRSA strains adapted to the CF lung after up to nine years of colonization was also affected by linezolid, ceftaroline and ceftobiprole. The dynamic analysis using the BioFlux^TM^ 200 system and ASM confirmed the activity of these three antimicrobial agents in limiting biofilm formation and showed that ceftaroline and ceftobiprole had a significantly greater effect than linezolid [40]. Finally, the activity of micronized tobramycin (4 μg/mL) and clarithromycin (200 μg/mL), alone or in combination, was also evaluated on 24-h-old and 12-day-old biofilms formed by six biofilm-forming strains of *S. aureus* (two MRSA and four MSSA) isolated from CF patients [137]. The main results obtained on these preformed biofilms were that: i) 12-day-old biofilms were systematically more resistant to antibiotics alone or in combination than 24-h-old biofilms; ii) the logarithmic decrease in colony-forming units (CFU) from antibiotic-treated biofilms was systematically greater with tobramycin (mean 1.07–5.31 and 0.31–4.44 CFU log_10_ decreases in 24-h-old and 12-day-old biofilms, respectively) than with clarithromycin (mean 0.52–3.74 and 0.17–1.22 CFU log_10_ decreases in 24-h-old and 12-day-old biofilms, respectively); iii) there was no influence from combining tobramycin with clarithromycin [137].

As not all anti-*S. aureus* therapeutic options have been yet evaluated for their anti-biofilm activity, such investigations have to be pursued to completely characterize the anti-biofilm potential and mechanisms of the different treatments against *S. aureus* since results might be helpful to guide the choice of the most effective therapy against CF airway infection.

### 5.4. Microbial Interaction

Among the bacterial genera described as having predatorial activity, the *Bdellovibrio bacteriovorus* species is a member of the *Oligoflexia* class found in the human gut microbiota and known to prey on Gram-negative bacteria. Iebba et al. showed that the *B. bacteriovorus* strain HD100 was also able to prey on a *S. aureus* isolate from a chronically mono-colonized CF patient. However, its mode of attack was however specific compared with Gram-negative prey as direct contact was observed during the whole predation process and three non-released bacteriolytic enzymes that remain to be characterized appeared to have a role in this predator-prey interaction [138]. *S. aureus* biofilms preformed during 24 h either statically (microtiter plate CV-straining) or under flow (BioFlux microfluidics system and electron microscopy biofilm vizualisation) were exposed to *B. bacteriovorus* in predatory assays. Epibiotic predation led to decreases in the amounts of preformed biofilm observed, both in static and dynamic settings, with a significant reduction in biofilm of 74% after 24 h of contact with the predator in the static assay and a 33% reduction after 14 h of contact, increasing to 46% after 20 h in the dynamic assay. The destruction of *S. aureus* cells seemed to occur by breaking down the bacterial wall and releasing the intracytoplasmic content of *S. aureus*. *B. bacteriovorus* was also shown to be a predator for CF *P. aeruginosa*, with an even higher reduction observed for preformed *P. aeruginosa* biofilm than *S. aureus*. Considering its presence in healthy human gut microbiota and its inability to infect mammalian cells, its ability to reduce established biofilms appeared of interest for in vivo applications during CF [138].

Surprisingly, our search criteria did not find any publications on bacteriophage anti-biofilm activity on CF *S. aureus* strains despite the growing interest in bacteriophage-mediated control of biofilm, based on the ability of these bacteriophages to penetrate existing biofilm and eliminate its structure [139]. This topic of interest warrants further investigations as the well-characterized staphylococcal bacteriophage, Sb-1, previously proposed as a promising tool to remove biofilms in other settings [140], was successfully used to treat a CF patient with chronic *S. aureus* colonization [141].

### 5.5. Miscellaneous

Ahonen et al. also studied the development of nitric oxide (NO)-releasing alginate oligosaccharides, considering NO’s anti-bacterial activity both on planktonic bacteria and biofilms and the need to decrease its toxicity through finely-controlled release [142]. Alginate oligosaccharides also have the ability to decrease mucus viscoelasticity. The study used two reference *S. aureus* strains only (MSSA ATCC 29,213 and MRSA ATCC 33,591) studied in ASM supplemented with 0.25% glucose and in two oxygen conditions for biofilm eradication assays with the aim of mimicking CF conditions more accurately. Biofilms grown in ASM for 48 h were challenged with NO-releasing alginates for 24 h under aerobic and anaerobic conditions and the results were compared to those obtained with tobramycin and vancomycin. The anti-biofilm effect of NO-releasing alginates was clearly demonstrated, particularly for Alg5-PAPA-DPTA/NO, with a 5-log reduction in biofilm viability observed after 24 h and alginates performed more efficiently than tobramycin and vancomycin in both oxygen conditions. As NO disrupts vital bacterial cell functions and structures through protein, DNA, and metabolic enzyme alterations, NO-releasing alginates display a broad-spectrum activity, including *P. aeruginosa* and *B. cepacia* in addition to *S. aureus*, making them promising candidates for future therapeutic options [142].

Pompilio et al. evaluated electrochemically synthesized silver nanoparticles (AgNP) formulation on a strong biofilm-forming *S. aureus* strain (*Sa*2) from a CF patient, showing the effectiveness of AgNPs against biofilm viability, with a dose-dependent effect and a maximum biofilm-killing rate of 98.2 ± 0.5% at 2x MIC [143]. These new formulations are active on biofilm formed by other pathogens like *P. aeruginosa* and shown to be non-toxic in in vivo studies on *G. mellonella* larvae. They require further investigation to elucidate their mode of action on *S. aureus* biofilms and their therapeutic potential for CF patients.

Physical stimulations, either electrical or magnetic, have also been applied to biofilms formed by *S. aureus* strains from CF patients [144,145]. In a context of increasing interest in the oral health of CF patients, Minkiewicz-Zochniak et al. analyzed the influence of low-intensity current on the ability of three CF *S. aureus* strains (one each among weak, moderate and strong biofilm formers) to form biofilm on titane (Ti-6Al-4V) and zirconium oxide biomaterials commonly used in dental implants [145]. Beside the implications on implant life cycle and potential local inflammation/infection processes, these biofilms are of importance as they may also represent a reservoir for both upper and lower airway infections in CF patients. Marked effects were noticed with zirconium compared with titanium. A low-amperage electrical current of 10 mA led to significant *S. aureus* biofilm reduction, affecting both adhesion (CV-staining evaluation) and *S. aureus* survival (live/dead fluorescence microscopy) as well as detaching biofilm-forming *S. aureus* from the biomaterial. Indeed, biofilm structure damage was visible after 10 min and attributed to an increase in the repulsive electrostatic forces between *S. aureus* and the biomaterial [145].

Altering the electrostatic interactions that might reduce *S. aureus* adhesiveness and the subsequent formation of biofilm was also the hypothesis drawn from the observation that applying a magnetic field of extremely low frequency to three *S. aureus* strains from CF patients (strong biofilm formers and multidrug resistant) significantly decreased biofilm formation and viability [144]. Indeed, selecting for a specific ionic channel (Ca^2+^, Cu^2+^, Fe^2+^/Fe^3+^, K^+^, Mg^2+^, Na^+^, Zn^2+^) showed that stimulating nearly all ions caused a significant reduction in *S. aureus* biofilm biomass formation (at least 25% compared with control) and in viability of the biofilm formed, suggesting that there were modifications in the attraction between the *S. aureus* surface, normally negatively-charged, and the positively-charged polystyrene surface used in this study. The high potential of such an intervention has been suggested to prevent biofilm formation or to eradicate biofilm on medical devices like nebulizers used by CF patients.

**Figure 3:**
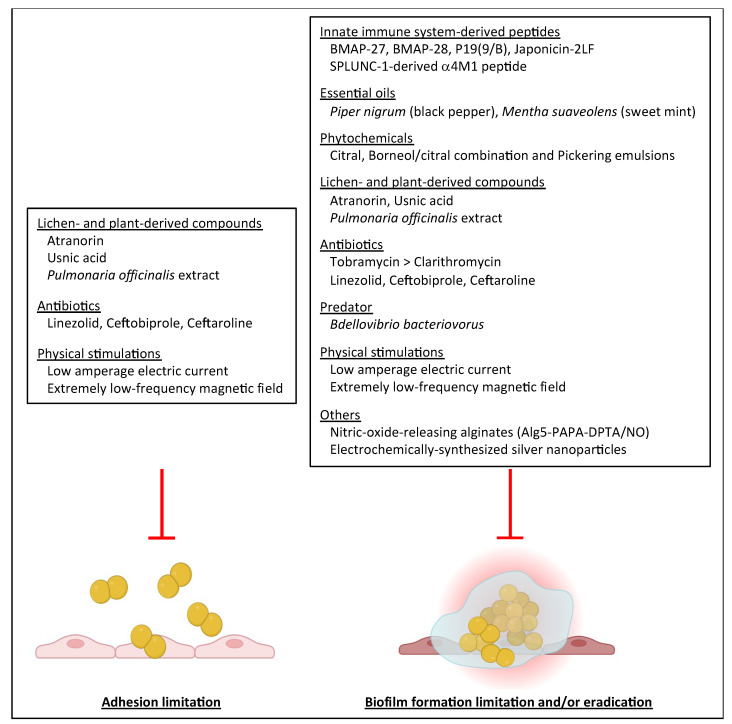
Presentation of anti-biofilm strategies shown to affect biofilm formation by *S. aureus* strains isolated from CF patients according to the two stages of biofilm formation specifically under study in the literature reviewed, i.e., adhesion and maturation/development (including biofilm formation and biofilm eradication).

A summary of the strategies limiting biofilm formation by *S. aureus* clinical isolates from CF patients is given in Figure 3 with respect to the stage of biofilm lifecycle under evaluation in the selected studies. 

In our review of the literature, we found no strategies with a specific action on the dispersal stage of biofilm but it is obvious that each affected stage of biofilm formation (adhesion, maturation and dispersal) will impact the subsequent ones (Figure 3). When available, mechanisms, as well as biological targets supporting the anti-biofilm activity of the strategies summarized in Figure 3 on clinical *S. aureus* strains isolated from patients with CF, were reported in the text. However, observational studies are the main studies performed in the CF context and affected targets by the anti-biofilm strategies reviewed in our manuscript remain largely unidentified in this context but also more generally in the literature.

## 6. Concluding Remarks

*S. aureus* is a major opportunistic pathogen in CF patients and biofilm production is a determining factor in the onset of persistent *S. aureus* respiratory infections in these patients. During CF, a broad panel of biotic and abiotic factors influences the different stages of biofilm development by *S. aureus* due to the specific, stressful conditions found in CF lungs. In this unique environment, *S. aureus* strains display specific features driven by bacterial adaptation and within-host evolution during persistence. Biofilm formation is one of these important traits representing a major obstacle, protecting *S. aureus* from host defenses and eradication attempts. Greater knowledge of the specific characteristics of biofilm formation by well documented *S. aureus* strains originating from CF patients is required as modulating or inhibiting biofilm formation is an important strategy for infection control and a target for the development of new therapeutic agents [125]. Although some of the current anti-biofilm approaches reviewed herein may be promising in the fight against *S. aureus* in CF patients, none of them has currently been currently evaluated during clinical trials. However, yet available results pave the way to further research that should address the more promising strategies for managing *S. aureus* infections in these patients but also their advantages and limitations. Indeed, biofilm eradicating strategies that kill bacteria independently of their physiological stage and are thus also active on persister cells within biofilm are of great interest [146]. The potential of combining diverse anti-biofilm strategies, i.e., biofilm eradicating agents, anti-adhesion agents, biofilm growth inhibitors, has also to be studied with special attention to be paid to synergy or antagonism that could establish between anti-biofilm agents but also between these agents and antibiotics. For some of the anti-biofilm agents under consideration herein like EOs, formulation development is also still required to enhance their efficacy and limit the impact of their usual dose-dependent effect on biofilm formation and, sometimes, toxicity before they could be considered as therapeutic options against biofilm-associated infections like CF [147]. Finally, several other approaches not yet investigated during CF may also be considered to enlarge the possibilities to counteract biofilm consequences [79,148].

## Data Availability

Not applicable.

## Supplementary Materials

The following supporting information can be downloaded from: https://www.mdpi.com/article/10.3390/ijms24010597/s1, Table S1: Data complementary to those given in Table 1 on patients, strains and biofilm evaluation for studies reviewed on biofilm formation by clinical strains of *S. aureus* isolated from patients with Cystic Fibrosis.
