# Supplementary material for: Biofilm Formation by Staphylococcus aureus in the Specific Context of Cystic Fibrosis
Source: Int J Mol Sci. 2022 Dec 29;24(1):597. doi: 10.3390/ijms24010597 (PMC9820612; doi:10.3390/ijms24010597)
Supplement: Supplementary file 1 [file ijms-24-00597-s001.zip › ijms-2076473-supplementary.pdf]

Table S1: Data complementary to those given in Table 1 on patients, strains and biofilm evaluation for studies reviewed on biofilm formation by clinical strains of *S. aureus* isolated from patients with Cystic Fibrosis.

CF patients			*S. aureus* strains			Biofilm formation		Ref.
**Mean (x) or median (Me) age [range]**	**Gender male (M) or female (F)**	**Other infecting species**	**Sequence types (ST) or *spa* types (n)**	**Specimens**	**Period of *S. aureus* collection**	**Method of study of biofilm formation**	**Molecular mechanism(s) involved in biofilm formation**	
x: 23.6 y [5-33] y	M: 9 F: 9	NA	ST228 (44), ST5 (5), ST247 (3), ST72 (1), ST235 (14), not typeable (26)	Sputum (88.2%), bronchial aspirates (9.6%), bronchial biopsy (2.2%)	1996 to 2006	CV	NA	[52]
NA	NA	NA	NA	Deep tracheal aspirates	2008 to 2010	CV	NA	[53]
x: 2.93 ±0.2 y	M: 103 F: 80	*P. aeruginosa, C. albicans, K. pneumoniae, E. cloacae, E. coli, S. marcescens, S. pyogenes*	NA	Sputum	March 2015 to August 2017	CV	NA	[55]
NA	NA	NA	NA	Sputum	NA	CV	NA	[35]
NA	NA	NA	8 different pulsotypes	Respiratory specimens	NA	CV	NA	[59]
<u>SCV positive patients</u>: Me: 24 y [5- 53] y <u>Normal phenotype positive patients</u>: Me: 23 y [1-48] y	<u>SCV positive patients</u>: M: 15 F: 13 <u>Normal phenotype positive patients</u>: M: 12 F: 17	<u>SCV positive patients</u>: *P. aeruginosa* (75%) <u>Normal phenotype positive patients</u>: *P. aeruginosa* (37.9%)	NA	Respiratory samples	NA	CV staining (w/v)	NA	[56]

Table S1 - *Cont.*

CF patients			*S. aureus* strains			Biofilm formation		Ref.
**Mean (x) or median (Me) age [range]**	**Gender male (M) or female (F)**	**Other infecting species**	**Sequence types (ST) or *spa* typing (n)**	**Specimens**	**Period of *S. aureus* collection**	**Method of study of biofilm formation**	**Molecular mechanism(s) involved in biofilm formation**	
Me: 27.9 y [19-45] y	M: 11<br>F: 3	*P. aeruginosa*: 6/14 patients chronically co-infected, not co-infected: 5/14 patients, new co-infection during study: 3/14 patients	Dominant *spa* types: t091, t1577, t008, t5430, t021, t166, t206, t034, t002, t617, t011, t003, t067	Sputum	1-y observational prospective study	CV	NA	[57]
x: 17.6 y [7-24] y	NA	Co-colonization with MSSA: 3/5 patients<br><br>No co-infection with *P. aeruginosa* and/or *B. cepacia*	ST22, ST247, ST8, ST291, ST72	Respiratory cultures	2-year prospective study: Feb. 2005 to Oct. 2007	CV	NA	[58]
x: 22.1 y [2-52] y	M: 16<br>F: 19	Co-colonization with *Sa* of different STs: 2/35 patients<br><br>Chronic colonization with *P. aeruginosa*: 21/35 patients	ST5 (32), ST8 (19), ST30 (3), ST34 (1), ST572 (3), ST4782 (1), ST4886 (1), ST5828 (1), ST5829 (1), ST239 (1)	Sputum	2007 to 2016	BRT® and BioFlux™ 200 microfluidic system	NA	[40]
NA	NA	NA	NA	Lung	NA	Continuous -flow culture bioreactors	NA	[60]
x: 19 y [17-22] y	NA	*P. aeruginosa*: 2/3 patients	ST8 (2), ST15 (2), ST5 (2)	Airway secretions	2008 to 2017	CV	Mutation in the *fakA* gene and in the *saeR* gene (up-regulation of SdrD adhesin)<br><br>Down-regulation of the *agr* regulon (overexpression of adhesins)	[61]

Table S1 - *Cont.*

CF patients			*S. aureus* strains			Biofilm formation		Ref.
Mean (x) or median (Me) age [range]	Gender male (M) or female (F)	Other infecting species	Sequence types (ST) or *spa* typing (n)	Specimens	Period of *S. aureus* collection	Method of study of biofilm formation	Molecular mechanism(s) involved in biofilm formation	
NA	NA	NA	t034 (4), t011 (2), t003 (2), t010 (2), t548 (2), t617 (2), t080 (2), t002 (2)	Sputum<br>Throat swabs	1994 to 2017	CV	NA	[62]
x: 9.7±1.2 y	NA	*P. aeruginosa*	t008 (36), t002 (29), t967 (1), t211 (2), t105 (1), t242 (4), t148 (1), t324 (2), t094 (1), t688 (2), t509 (1), t895 (2), t062 (2), t1577 (2), t304 (1), t024 (3), t216 (2), t1183 (2), t622 (4)	Oropharyngeal swabs (52)<br>Sputum (41)<br>Bronchoalveolar lavage (5)	2006 to 2014	CV	NA	[63]
x: 21.8 y [6.3–42.3] y	NA	*P. aeruginosa*	ST1 (4), ST5 (4), ST7 (2), ST8 (4), ST15 (10), ST25 (4), ST26 (2), ST30 (10), ST34 (2), ST45 (2), ST101 (6), ST398 (4), ST509 (2), ST619 (2)	Sputum	since 1994…	CV	NA	[64]
Me: 24 y [5-34] y	M: 7<br>F: 1	*P. aeruginosa*: 3/8 patients	ST25, ST188, ST5, ST30, ST7, ST1909	Nasal<br>Throat<br>Sputum	NA	CV / confocal microscopy	5bp-deletion (TATTT) between *icaR* and *icaA* affecting binding of repressor of biofilm (rob)<br><br>Not all strains with 5 bp-deletion were mucoid (compensatory mutations)	[65]

Table S1 - *Cont.*

CF patients			*S. aureus* strains			Biofilm formation		Ref.
**Mean (x) or median (Me) age [range]**	**Gender male (M) or female (F)**	**Other infecting species**	**Sequence types (ST) or *spa* typing (n)**	**Specimens**	**Period of *S. aureus* collection**	**Method of study of biofilm formation**	**Molecular mechanism(s) involved in biofilm formation**	
Patients with mucoid isolates: Me: 29 y [14-39] y<br><br>Patients without mucoid isolates: Me: 17 y [9-24.5] y	Patients with mucoid isolates: M: 5 F: 2<br><br>Patients without mucoid isolates: M: 36 F: 38	*P. aeruginosa:* 4/7 Patients with mucoid isolates and 24/74 Patients without mucoid isolates	Mucoid isolates: t493, t189, t002, t267, t091, t893, t618<br><br>Non-mucoid isolates: t091, t189, t002, t267, t091, t003, t011	Throat Sputum	Beginning of March 2017 to the end of May 2017	CV	Not all mucoid strains carried 5 bp-deletion<br><br>Not all mucoid strains were biofilm producers	[66]
Me: 9.5 y	NA	NA	ST5 (7), ST8 (5), ST12 (2), ST15 (3), ST30 (7), ST45 (2), ST59 (4), ST83 (1), ST87 (2), ST121 (1), ST149 (1), ST221 (1), ST225 (2), ST461 (1), ST1159 (2), ST1498 (1), not typeable (8)	Sputum Throat	NA	CV	Cobalt transporter ATP binding domain (*cbiO*): required for biofilm formation<br><br>Copper ions (Cu^{2+}): cofactors to mediate biofilm formation<br><br>Iron ions (Fe^{3+}): decreased the ability to form biofilm	[67]
Paediatric patients	NA	NA	NA	NA	NA	CV	Sub-inhibitory concentrations of bile (sodium cholate and sodium deoxycholate bile acids) enhanced biofilm formation	[68]

Table S1 - *Cont.*

CF patients			*S. aureus* strains			Biofilm formation		Ref.
**Mean (x) or median (Me) age [range]**	**Gender male (M) or female (F)**	**Other infecting species**	**Sequence types (ST) or *spa* typing (n)**	**Specimens**	**Period of *S. aureus* collection**	**Method of study of biofilm formation**	**Molecular mechanism(s) involved in biofilm formation**	
x: 37.7 y [27-45] y	M: 2 F: 1	*P. aeruginosa*, MSSA, *H. parainfluenzae*, *H. influenzae*, *E. coli*	ST105 (6), ST109 (3), ST5 (3)	Sputum Blood Bronchoalveolar lavage	2013 to 2017	CV	Mutations and altered expression of *dacA* and *gpdP* genes: *dacA* encodes a deadenylate cyclase required for the synthesis of cyclic-di-AMP (c-di-AMP) *gpdP* encodes a phosphodiesterase responsible for c-di-AMP hydrolysis<br><br>Mutations in *saeR*<br><br>Increased expression of *gapR*, *zwf*, *sdh* and *mqo1*<br><br>Decreased expression of *gltA*<br><br>Increase in *fumC* expression +++ (malate dehydratase increasing fumarate concentrations)	[69]

CV: crystal violet staining (w/v); MSSA: methicillin-susceptible *Staphylococcus aureus* (*Sa*); NA: not available; SCV: small colony variant; y: years; BRT®: BioFilm Ring test®; fakA: fatty acid kinase A gene; *saeR*: *Staphylococcus* exoprotein expression protein R gene.
